# Factors associated with full childhood vaccination coverage among young mothers in Northern Nigeria

**DOI:** 10.11604/pamj.2024.47.4.37517

**Published:** 2024-01-04

**Authors:** Alabi Matthew Ayodele, Motunrayo Idiat Fasasi, Obiora Rejoice Uche, Nwankwo Gideon Ikemdinachi, Ukwu Henry Ugochukwu

**Affiliations:** 1 Programmes Division, Population Council Abuja, Abuja, Nigeria,; 2 Department of Nursing, Fountain University Osogbo, Osogbo, Nigeria,; 3 Department of Health Promotion and Community Health, American University of Beirut, Beirut, Lebanon,; 4 Department of Health Management and Policy, American University of Beirut, Ras Beirut, Lebanon,; 5 Department of Computer Engineering, Amsterdam Tech, Amsterdam, Netherlands

**Keywords:** Full immunization coverage, young mothers, immunization, Northern Nigeria

## Abstract

**Introduction:**

wide regional variation in immunization coverage still persists in Nigeria. Full Immunization Coverage (FIC) for more than 80% of all states in the northern region is lower than 40% relative to their southern counterpart. Studies focusing on young women in the north remain sparse, despite the high prevalence of early marriage and poor health-seeking behavior. This study examines FIC among young women in northern Nigeria.

**Methods:**

we performed a secondary analysis of the 2013 and 2018 Nigeria Demographic and Health Survey on 1,198 women of children aged 12-23 months in 2013 and 405 in the 2018 dataset. Analysis was limited to young women 15-24 years, residing in Northern Nigeria. We used logistics regression to predict factors associated with FIC.

**Results:**

the proportion of fully immunized children was low, at 11% in 2013 and 18% in 2018. The coverage for most vaccines was low, except for the oral polio vaccine. The children of mothers who had health card [(aOR=18.1,95% C.I (8.1-40.7)], in 2013 and 2018 [(aOR=12.7, 95% C.I (5.9-27.1)], attended ANC [(aOR=8.6, 95% C.I (2.4-30.9)] in 2013 and had facility delivery [(aOR=2.0, 95% C.I (1.0-4.1)] in 2018 were more likely to be fully immunized.

**Conclusion:**

the study found FIC among children of young women in Northern Nigeria was abysmally low. Ownership of health care, antenatal attendance, and facility delivery significantly predicted the odds of FIC. These findings suggest the need for approaches that remove barriers to good health-seeking behavior, especially among young mothers in Northern Nigeria.

## Introduction

Nigeria is the most populous country in Africa with more than 200 million people cutting across different religious and ethnic groups [1]. The country is divided into north and south, with sharp disparity in health and sexual and reproductive health indicators, at the disadvantage of the northern region [2]. Full Immunization Coverage (FIC) among children 12-23 months for more than 80% of all the states in the northern region of Nigeria as of 2018 was lower than 40%, relative to that of children of mothers from the southern region of the country, where some states have reported over 65% coverage. Also, there is limited knowledge about full child coverage immunization coverage among young mothers especially in the northern region of the country characterized by low age at marriage, low childbearing age, and poor health-seeking behavior [2]. This is despite the fact that childhood immunization serves as a preventive measure against diseases including diphtheria, pertussis, tetanus, measles, and tuberculosis, thereby preventing the deaths of between 2-3 million children yearly [3]. Likewise, immunization has been widely accepted as one of the most cost-effective public health measures with huge direct and social benefits [4,5].

Remarkably, the global trend in childhood vaccination coverage has shown notable improvement. For instance, the coverage for diphtheria-tetanus pertussis (DPT) and measles has increased from 72% to 86% for DPT and 72% to 85% for measles between the years 2000 and 2016 [4]. However, despite this improvement, wide variation exists between developed and developing countries of the world in terms of complete child immunization coverage. Among infants 0-11 months, global DPT 3 coverage in 2019 was 26% in 46 sub-Saharan African countries and 89% in 36 high-income countries [6]. For the majority of these high-income countries, the majority of the infectious diseases have been contained and, in most cases, eliminated through effective vaccination programs evidenced by a high level of vaccination coverage and decline in reported cases of vaccine-preventable disease [7].

In contrast, despite the gains and cost-effectiveness of vaccination in preventing childhood morbidity and mortality, childhood vaccination coverage (putting into consideration infants that have received all the recommended doses by the time they are age 12 months and above) in Nigeria remains abysmally low. In the year 2018, Nigeria alone accounts for more than one-quarter of the global unvaccinated children [8]. Similarly, as of 2018, less than one-quarter of children 0-11 months were fully immunized [2]. Recent statistics revealed the national coverage for full vaccination (all first-year antigens) among children 12-23 months in Nigeria is 35.6%, with sharp regional variation. The highest coverage in the northern region was reported in the north-central at 32.4%, while the highest coverage in the south was reported in the southeast at 57.3% [9]. All these coverages are far below the Global Vaccine Action Plan (GVAP) target of at least 90% [10].

However, several factors have been implicated in low vaccination coverage, especially in the northern regions of the country. Studies examining factors associated with child vaccination have reported several findings including maternal level of education [11-13], place of residence, wealth index, and postnatal care [14], religion [15], and insecurity [16,17] among others. The majority of these studies have largely focused on women of reproductive age 15-49 years. Recent studies [18-20] have reported factors including maternal level of education, place of residence, delivery place, exposure to mass media, partner level of education, wealth index, and postnatal attendance were all significantly associated with full child immunization coverage. Likewise, ownership of health cards, good knowledge of the importance of vaccination, and mothers receiving tetanus toxoid vaccination during ANC [21,22] have been reported to be associated with full child vaccination status.

However, virtually all these studies have focused mainly on women of reproductive age, while studies examining coverage among young women between the ages of 15-and 24 years in the north remain sparse, despite the high prevalence of child marriage and low age at bearing [23]. Also, most of these teenage mothers are often faced with poor health-seeking behavior relative to older women [24-29]. Studies have grouped these barriers to adolescent health-seeking behavior into individual-level factors; including poor knowledge and attitude towards reproductive health service; social factors which include the influence of parents, community, religious factors, stigma from the society, and financial constraints and institutional factors namely inconvenient operating hour of health facilities, poor attitude of health personnel towards adolescents sexual and reproductive health among others [25,26]. Recent studies conducted in Northern Nigeria have identified barriers to poor health-seeking behavior among adolescent mothers to include lack of privacy and confidentiality, perceived negative attitude of healthcare workers, financial constraints and services not adolescent-friendly, proximity to health facilities, and long waiting hours at health facility [30-32].

Therefore, the need to intensify efforts toward achieving FIC in Nigeria, particularly in the northern region, is highly imperative. Besides addressing the immunity gap, it will help address the issue of re-emergence of vaccine-preventable diseases. This is important given the re-emergence of polio in some Northern Nigerian states reported in 2021 shortly after Nigeria was declared polio-free by WHO in 2020 [33]. This re-emergence was attributed to low immunization coverage, and poor sanitation, which might be fueled in part by a lack of sustainability in gains and efforts towards eradication of vaccine-preventable diseases in the northern region [34]. Hence, the government and relevant stakeholders must sustain the gains in childhood immunization coverage to achieve long-term gains and prevent a re-emergence. Thus, the specific objective of this study is to examine full immunization coverage among young women in 15-24 years in Northern Nigeria.

## Methods

**Study design:** the study used data from the two most recent Nigeria Demographic and Health Survey (NDHS) 2013 and 2018, a population-based cross-sectional survey.

**Study design:** the study used data from the two most recent Nigeria Demographic and Health Survey (NDHS) 2013 and 2018, a population-based cross-sectional survey.

**Setting:** the study performed a secondary analysis of the two most recent Nigeria Demographic and Health Survey (NDHS 2013, 2018) datasets. The NDHS is a nationally representative survey of women in their reproductive ages 15-49 years across the 36 states of Nigeria and the Federal Capital Territory, Abuja. Data collection for the 2013 NDHS was collected between December 2012 and January 2013, while data for the 2018 NDHS was collected between August and December 2018 (more details on recruitment among others have been reported elsewhere in the full report).

**Study participants:** data were collected from women aged 15-49 years who had given birth to at least one live birth five years period preceding the survey, in addition to men 15-59 years. More detailed information about the sampling technique used for the survey has been presented in the final report. The study collected data using a questionnaire covering different health areas including maternal and child health, infant and child mortality, fertility, marriage and sexuality, family planning, and contraceptive use among others.

**Study size:** for this current study, the sample was restricted to young women 15-24 years who are currently married and living with their partner, have given birth to at least a child in the last five years preceding the survey, and residing in the northern region of the country. The actual national sample size of women in this age group with children aged 0-4 years across all the thirty-six states of the federation and the Federal Capital Territory was: year 2013 (28,596) and 2018 (30,713). However, restricting the dataset to a sample of interest as defined above, the final weighted sample size was reduced to 1,198 women and 405 women of children aged 12-23 months for the 2013 and 2018 NDHS datasets respectively. At every point of analysis, data were weighted using the weighting variable (v005) generated by the DHS survey to restore the national representativeness of the data. The choice of the age group 12-23 months was because it is expected that a child should have completed immunization at that age. Information about child vaccination was collected through vaccination cards and the mothers´ verbal reports.

### Measurement of variables

**Outcome variable:** the outcome variable is full vaccination coverage, defined as children 12-23 months who had received all the recommended basic vaccines in appropriate doses. We adopted NDHS definition based on WHO recommendation that a child can be considered fully vaccinated if the child receives all the eight vaccines namely the birth dose of BCG, three doses of oral polio vaccine excluding OPV dose given at birth, three doses of DPT-HepB-Hib (DPT 1,2,3) and one dose of measles vaccine [35]. Thus, fully vaccinated children were coded as 1, otherwise 0.

**Explanatory variables:** explanatory variables used in this study are individual-level variables; the age of respondent (15-19 years and 20-24 years), level of education (no formal education, primary, secondary, and tertiary), and wealth status were originally categorized into five quantiles by the DHS and derived from the measurement of ownership of household items including car, radio, television, toilet facilities, water source and type of roofing. These measures have been used by the World Bank to categorize households into poverty levels based on principal component analysis [36]. However, for this study, we re-categorize the wealth index into poor, middle, and rich for ease of interpretation. Region (northeast, northwest, and north central), religion (Christian versus Islam), place of residence (rural versus urban), exposure to mass media, partner´s level of education (no education, primary, secondary, and tertiary), and autonomy (dichotomized into a woman has autonomy versus no autonomy). Four variables were used to generate a composite variable on autonomy, namely: a person who usually decides on respondents´ healthcare; a person who usually decides on large household purchases; a person who usually decides whether the respondent can visit family or relatives, and the person who usually decides on how partner earnings is spent. Each of the variables were recorded into individual decisions or joint decisions. The second level of explanatory variables used were termed institutional variables namely ownership of child vaccination card by the woman and whether distance to a health facility is a problem or not. The third level of explanatory variables are mothers´ health seeking measured using antenatal attendance by the woman during the last pregnancy, a number of ANC visit categorized into less than 4 visits and 4+ visits, place of delivery (facility versus non-facility delivery), and postnatal check for the baby after birth.

**Statistical methods:** the analysis of data was performed at three levels, namely univariate, bivariate, and multivariate. At the univariate level, frequency count and percentages were performed on both the outcome and explanatory variables. For the bivariate and multivariate levels, binary logistics was performed. Bivariate logistics regression was performed using each of the explanatory variables with the outcome variable. Thus, only variables that were significantly (at 5% alpha level) associated with the outcome variable at the bivariate level were included at the multivariate level. At the multivariate level, binary logistic regression was performed with odds ratios, and 95% confidence interval was reported. Statistical significance was considered at p<0.05. All missing cases were excluded from the analysis. The choice of binary logistic regression was due to the dichotomous nature of the outcome variable, coded as 1 for full vaccination status and 0, for incomplete vaccination. Two models were constructed for each of the data point (2013 and 2018). Model one included individual-level variables, while model two combined both individual, institutional, and maternal health-seeking behaviour variables. Data was analyzed using Stata 15 software. Due to the complex nature of the DHS design, the ´svy´ command was employed during analysis to adjust for clustering and sampling weight to ensure the data is nationally representative.

**Ethical consideration:** the protocol used for the Nigeria Demographic and Health Survey has been reviewed and approved by the statutory regulatory, the National Health Research Ethics Committee (NHREC), and the ICF Institutional Review Board. In addition, all the questionnaires were translated into the three major languages in the country namely Hausa, Yoruba, and Igbo. However, to use the data set, approval was obtained from measure DHS after registering at the website, indicating the purpose for which the dataset will be used.

## Results

**Socio-demographic characteristics of participants:** Table 1, Table 2, Table 3 present the result of the descriptive analysis of respondent´s socio-demographic characteristics, institutional variables, and maternal health-seeking behavior and the outcome variable, showing vaccination coverage. More than three-quarters (74% vs. 82%) of the young women in the 2013 and 2018 surveys were in the age group 20-24 years, (67% vs. 60%) in the year 2013 and 2018 had no formal education, while (52% vs. 49%) of the young women were residence in the northwest region of the country. According to location, (82% vs. 76%) were residing in rural areas, while (64% vs. 61%) belonged to the poor household wealth quintile. The percentage of women exposed to mass media (51% vs. 47%), with autonomy (24% vs. 25%) in the 2013 and 2018 samples, respectively. A greater percentage of the women´s partners had no education (57% vs. 44%). Findings from the institutional and health-seeking behavior of the women indicate; (73% vs. 39%) of the women had health card for their children, (63% vs. 37%) considers distance to health facilities as a problem. Regarding health-seeking behavior (48% vs. 68%) visited antenatal care during the last pregnancy. Similarly, (33% vs. 42%) of the women had 4+ ANC during the last pregnancy, while (22% vs. 26%) of the young mothers had facility delivery.

**Table 1:** background characteristics of respondents

Socio-demographic variables	2013 n=1198	%	2018 n= 405	%
**Age groups**				
15 - 19	305	25.5	74	18.3
20 - 24	893	74.5	330	81.7
**Level of education**				
No formal education	808	67.4	241	59.7
Primary	187	15.6	60	14.9
Secondary	192	16.0	97	24.0
Higher education	11	1.0	6	1.4
**Region**				
North-central	245	20.5	98	24.2
North-east	328	27.3	107	26.5
North-west	625	52.2	199	49.3
**Residence**				
Rural	986	82.3	308	76.0
Urban	212	17.7	97	24.0
**Religion**				
Christianity	158	13.2	337	83.2
Islam	1016	84.8	68	16.8
Traditionalist	24	2.0	-	-
**Wealth quintile**				
Poor	770	64.3	248	61.2
Middle	234	19.5	85	21.0
Rich	194	16.2	72	17.8
**Exposure to mass media**				
Yes	607	50.7	189	46.8
No	591	49.3	216	53.2
**Autonomy status**				
Has autonomy	285	23.8	101	24.8
No autonomy	913	76.2	304	75.2
**Partner’s education level**				
No education	679	56.7	179	44.1
Primary	149	12.4	58	14.3
Secondary	278	23.2	125	30.9
Higher	92	7.7	43	10.7

**Table 2:** institutional variables and maternal health-seeking behaviour

Health card ownership	2013 n=1,198	%	2018 n=405	%
The child has a health card	874	72.9	156	38.6
No health card	324	27.1	249	61.4
**Distance to the health facility**				
Seen as a problem	757	63.1	149	36.8
Not a problem	441	36.9	256	63.2
Antenatal attendance				
Yes	579	48.4	273	67.5
No	555	46.3	125	30.8
Missing/don’t know	64	5.3	7	1.7
**Number of ANC attendance**				
Less than 4 visits	183	15.3	105	25.9
4+ visits	396	33.0	168	41.6
Unknown/missing	619	51.7	132	32.5
Place of delivery				
Health facility	261	22.0	107	26.3
Non-health facility	932	78.0	298	73.7
Unknown	5	0.4	-	-
**Post-natal check for baby**				
Yes	208	17.4	57	14.2
No	942	78.6	340	84.1
No response	48	4.0	7	1.7
ANC; Antenatal care (ANC)				

**Table 3:** vaccination coverage among children 11-23 months

Received BCG	2013 n=1,198	%	2018 n=405	%
Yes	340	28.3	209	51.6
No	858	71.7	196	48.4
**Polio 0**				
Yes	331	27.6	145	35.8
No	867	72.4	260	64.2
**Received DPT1**				
Yes	342	28.6	202	49.8
No	856	71.4	203	50.3
**Received DPT2**				
Yes	283	23.6	167	41.2
No	915	76.4	238	58.8
**Received DPT3**				
Yes	212	17.7	128	31.6
No	986	82.3	277	68.4
**Polio 1**				
Yes	806	67.3	271	66.8
No	392	32.7	134	33.2
**Polio 2**				
Yes	718	60.0	238	58.8
No	480	40.0	167	41.2
**Polio 3**				
Yes	583	48.7	169	41.8
No	615	51.3	236	58.1
**Measles**				
Yes	294	24.6	144	35.5
No	904	75.4	261	64.5
**Child’s vaccination status**				
Full vaccination	126	10.5	75	18.4
Incomplete vaccination	1072	89.5	330	81.6

BCG: Bacillus Calmette-Guérin, DPT: diphtheria pertussis tetanus

**Outcome data:** the result on child vaccination coverage showed only 11% of the children aged 12-23 months were fully immunized in 2013, while 18% of the children were fully immunized in the year 2018. This represents a 7% increase in the proportion of fully immunized children aged 12-23 months among young women 15-24 years for the five-year period (2013-2018). Regarding coverage rates for birth antigens, only 28% of the children received BCG birth doses in 2013 and 52% in 2018, while 28% of the children received OPV 0 in 2013 and 36% in 2018. For other antigens, 29% versus 50% of the children received DPT 1 in 2013 and 2018, respectively. For DPT 2 (24% vs. 41%) and DPT 3 (18% vs. 32%) during the year 2013 and 2018 respectively. For oral polio vaccine, OPV 1 (67% vs. 67%); OPV 2 (60% vs. 59%) and OPV 3 (49% vs. 42%). For measles vaccine, (11% vs. 18%) during the year 2013 and 2018 respectively.

**Multivariate:** for the year 2013, the result of model one examining the effect of maternal characteristics on the odds of full child vaccination coverage showed maternal level of education, religion, and household wealth were significantly associated with the odds of full child immunization. Women with tertiary education (OR=5.03, p<0.05), belonging to the middle (OR=3.02, p<0.05) and rich (OR=3.97, p<0.05) household wealth quintile demonstrated high odds of having full vaccination for their children. Also, partners' level of education was significantly associated with the odds of full child immunization among young women. On the other hand, women affiliated to the Islam religion demonstrated lower odds (OR=0.27, p<0.05) of full child vaccination. However, adjusting for maternal characteristics, institutional variables, and maternal health-seeking behavior; only religion, ownership of child vaccination card, and antenatal care attendance were significantly associated with the odds of full child vaccination. While women affiliated to the Islam religion demonstrated lower odds (OR=0.28, p<0.05) of full child vaccination coverage, children of women with child health cards (OR=18.1, p<0.05), who attended antenatal care during the last pregnancy (OR=8.61, p<0.05) demonstrated higher odds of being fully vaccinated.

Moreover, findings from the analysis of the 2018 data showed none of the maternal characteristic variables (model one) significantly predicted the odds of full child vaccination. In contrast, the adjusted odds ratio (model two) showed ownership of the child's health card and place of delivery significantly predicted the odds of full child vaccination. Children of young women with health cards demonstrated higher odds (OR=12.7, p<0.05) of being fully vaccinated relative to those without a health card. Also, young women who delivered at the health facility demonstrated higher odds (OR=2.05) of having their child fully vaccinated (Table 4).

**Table 4:** binary logistic regression analysis of factors associated with full child vaccination

	NDHS 2013 [n=1,198]				NDHS 2018 [n=405]			
Age groups	Model 1		Model 2		Model 1		Model 2	
	OR	95% C.I.	OR	95% C.I.	OR	95% C.I.	OR	95% C.I.
15 - 19	RC		RC		RC		RC	
20 - 24	1.16	0.66-2.00	0.76	0.39-1.46	1.19	0.58-4.10	1.14	0.50-2.59
**Level of education**								
None	RC		RC		RC		RC	
Primary	0.85	0.44-1.65	0.66	0.31-1.38	1.89	0.88-4.10	1.21	0.50-2.91
Secondary	1.86	0.99-3.49	1.47	0.72-3.03	1.70	0.76-3.78	0.93	0.37-2.34
Higher education	5.03*	1.29-19.7	2.58	0.60-11.1	4.54	0.63-32.6	3.22	0.33-31.2
**Region**								
North-central	RC		RC		RC		RC	
North-east	1.46	0.85-2.52	1.33	0.70-2.52	1.11	0.54-2.28	1.32	0.56-3.12
North-west	1.00	0.56-1.81	1.49	0.74-2.98	0.77	0.36-1.65	1.10	0.45-2.66
**Residence**								
Urban	RC		RC		RC		RC	
Rural	0.84	0.48-1.47	1.04	0.54-2.00	0.68	0.34-1.34	0.77	0.35-1.70
**Religion**								
Christianity	RC		RC		RC		RC	
Islam	0.27*	0.15-0.48	0.28*	0.14-0.56	0.55	0.27-1.10	0.54	0.24-1.20
**Wealth quintile**								
Poor	RC		RC		RC		RC	
Middle	3.02*	1.72-5.30	1.76	0.90-3.43	1.05	0.51-2.15	0.92	0.40-2.11
Rich	3.97*	1.86-8.45	1.64	0.68-3.98	1.56	0.69-3.54	1.76	0.67-4.60
**Media exposure**								
No	RC		RC		RC		RC	
Yes	1.13	0.69-1.86	1.21	0.66-2.21	1.16	0.65-2.06	1.04	0.53-2.03
Autonomy status								
No autonomy	RC		RC		RC		RC	
Has autonomy	1.07	0.67-1.68	1.00	0.58-1.72	0.78	0.43-1.42	0.53	0.27-1.07
**The child has a health card**								
No			RC				RC	
Yes			18.1*	8.1-40.7			12.7*	5.9-27.1
**Distance to health facilities is a problem**								
No			RC				RC	
Yes			0.67	0.33-1.34			1.33	0.67-2.63
**Antenatal attendance**								
No			RC				RC	
Yes			8.61*	2.39-30.9			1.91	0.73-4.98
Place of delivery								
Outside facility			RC				RC	
Health facility			1.26	0.73-2.16			2.05*	1.03-4.11
**Post-natal check for baby**								
No			RC				RC	
Yes			1.07	0.64-1.78			1.15	0.55-2.38
**Partner education status**								
None	RC		RC				RC	
Primary	2.38*	1.20-4.72	1.47	0.65-3.30			1.15	0.41-3.19
Secondary	1.61	0.85-3.09	0.73	0.34-1.53			0.78	0.32-1.90
Higher	1.52	0.69-3.36	0.61	0.24-1.53			0.87	0.26-2.94

** RC=Reference Category; OR=Odds Ratio, C.I. = Confidence Interval, NDHS=Nigeria Demographic and Health Survey

## Discussion

The study found low vaccination coverage for birth antigens (BCG and OPV 0) among children of young women in Northern Nigeria. While findings showed improvement in coverage rate for birth antigens between 2013 and 20118, coverage for BCG remains at slightly more than 50%, while coverage rate for OPV 0 remains less than 50%. Regardless of the survey period, coverage was highest for the polio 1 vaccine. Measles coverage regardless of the survey year was less than 50%. Overall, full vaccination coverage for children 12-23 months was very low - below 20% among young women. This no doubt has implications on the vaccination coverage target, especially among children of young women from the northern part of the country who had hitherto constituted the region for low vaccination coverage in the country [23]. We found ownership of child health card, antenatal attendance, and facility delivery as important predictors of full child vaccination. Regardless of the data point, children of young women with health card demonstrated higher odds of being fully vaccinated. This corroborates findings from previous studies [21,22]. This implies children of women with health card are more likely to be fully vaccinated relative to those without child´s health card. Ownership of child health card will no doubt allow for effective tracking of child vaccination status for both the mother and the healthcare provider. In addition, mothers who lost their card should be encouraged by the health providers to get replacements with minimal or no cost. This will ensure that women who lost their child´s immunization card do not hide under the umbrella of not having an immunization card for not presenting their child for subsequent doses because of its likely impact on full immunization coverage.

The outcome of our study showed the use of health service among young women contributes positively to child vaccination status. For instance, children of women who attended antenatal were more likely to be fully immunized relative to children of young women who did not attend antenatal during pregnancy. Similar findings have been previously reported [37-39]. No doubt, attendance of antenatal by these young women would have afforded them the opportunity to access adequate information regarding the benefits of routine childhood immunization and the dangers of not vaccinating their children. This will ultimately help them in making informed health decisions. Children of women who had facility delivery are twice more likely to be fully vaccinated relative to those who delivered at home. This has been corroborated by previous studies [20,40]. Women who deliver at the health facility will have the opportunity to vaccinate their child after birth and also receive adequate information about subsequent immunization schedules for their children. This is very important as previous studies [31] have shown antenatal care visit and family planning to be very strong factors for using healthcare services among adolescent mothers. This study shows the importance of child health care in the quest for achieving full child vaccination. Despite the low proportion of young women in 2018 who had health care for their children, ownership of a health card was significantly associated with higher odds of full child vaccination. Also, the importance ANC attendance and facility delivery cannot be over-emphasized, especially among young women who are often faced with difficulty in accessing healthcare for several factors including culture, religion among others [23,41,42]. Furthermore, the significant effect of religion on full child vaccination is worth noting considering that Islam is the predominant population of the region and exert strong influence on all aspects of their life. In addition, this echoes the need for the continuous inclusion of religious leaders as critical partners in strategies for communicating information regarding child immunization because of their influence on their followers. The positive impact of these Islamic leaders in eliminating poliomyelitis in Africa (Nigeria inclusive) and India have been previously reported [43-45]. These findings suggest the need for approaches that remove barriers to good health-seeking behaviour especially among young mothers in Northern Nigeria in addition to equipping these young women with accurate and comprehensive sexual and reproductive health information and strengthening institutional factors such as antenatal attendance and family planning services [31] if the region is going to make a substantial improvement in child vaccination coverage.

**Limitation:** this study is limited by the use of cross-sectional secondary data and poses some limitation in terms of establishing causal relationships. Also, the sample size for the 2018 data set was relatively smaller than that of 2013. The limited sample size particularly for the 2018 data set suggest some form of limitation regarding the extent to which the result can generalize to the general population.

## Conclusion

The study has further re-emphasized the importance of institutional factors and good maternal health-seeking behavior in achieving full childhood vaccination coverage. An institutional factor that the government and relevant stakeholders must pay important attention to in the quest to achieve an improved child vaccination program is the availability of child health cards for mothers. This health card generally contains information about the immunization schedule for the child from birth to the very last dose, in addition to other vital health information. Ensuring that all caregivers have this health card without stress will allow both the caregivers and the health provider to monitor and track the immunization schedule for the child. Holistic review of this health card by the health provider will go a long way in ensuring that children are well monitored, and tracked, and also promote good defaulter tracking among the caregivers. Regarding maternal health-seeking behavior, a policy that will strengthen antenatal care delivery and promotion of facility delivery will no doubt contribute significantly to achieving improved child vaccination coverage among teenage mothers in the northern region of the country in particular and Nigeria as a whole. Hence, approaches to remove barriers to antenatal care and encourage facility deliveries among women in Northern Nigeria should be prioritized.

### What is known about this topic

- *Full child vaccination coverage is abysmally low in Nigeria, with huge regional variation;*
- *Factors associated with full child vaccination among older women have been well researched in Nigeria*.

### What this study adds

- *Full vaccination coverage is abysmally low among young mothers; coverage for most vaccines was low and highest for OPV 1;*
- *Ownership of child health care, antenatal care attendance, and facility delivery were significantly associated with full vaccination coverage;*
- *Institutional factors are important predictors of full child vaccination coverage among young mothers*.
